# Small Activating RNA Restores the Activity of the Tumor Suppressor HIC-1 on Breast Cancer

**DOI:** 10.1371/journal.pone.0086486

**Published:** 2014-01-28

**Authors:** Feng Zhao, Shengli Pan, Yan Gu, Shanyu Guo, Qiancheng Dai, Yingyan Yu, Wei Zhang

**Affiliations:** 1 Department of Surgery, The Ninth People's Hospital of Shanghai Jiao Tong University, School of Medicine, Shanghai, China; 2 Department of Surgery, Shanghai Ruijin Hospital of Shanghai Jiao Tong University, School of Medicine, Shanghai, China; Baylor College of Medicine, United States of America

## Abstract

HIC-1 is a gene that is hypermethylated in cancer, and commonly downregulated in human breast cancer. However, the precise mechanisms and molecular pathways regulated by HIC-1 remain unclear. We assessed HIC-1 expression on a tissue microarray containing 80 cases of breast cancer. We also analyzed its biological function by restoring HIC-1 expression using 5-aza-2′ deoxycytidine (5-CdR) and small-activating RNAs for the reversal of HIC-1 tumor suppressive effects on MCF-7 and MDA-MB-231 cell lines. An Agilent Q44h global expressing microarray was probed after restoring the expression of HIC-1. Data demonstrated that HIC-1 expression was reduced significantly in breast cancer tissues. HIC-1 immunohistochemistry resulted in mean staining scores in cancer tissue and normal ductal epithelia of 3.54 and 8.2, respectively (*p*<0.01). 5-CdR partially reversed HIC-1 expression, and modulated cell growth and apoptosis. dsHIC1-2998, an saRNA, showed activating efficacy in breast cancer cells. A group of differentially expressed genes were characterized by cDNA microarray. Upon saRNA treatment, genes upregulated included those involved in immune activation, cell cycle interference, the induction of apoptosis, anti-metastasis, and cell differentiation. Downregulated genes included oncogenes and those that play roles in cell invasion, cell growth, and cell division. Our findings may provide valuable resources not only for gene functional studies, but also for potential clinical applications to develop novel drug targets.

## Introduction

Breast cancer is one of the most common malignancies worldwide, and severely influences public health. Currently, operation combined with chemotherapy or targeted therapy remains the major strategy for breast cancer treatment. Targeted therapeutic strategies include the use of epidermal growth factor receptor inhibitors, anti-angiogenic agents, cell cycle inhibitors, apoptosis promoters, and matrix metalloproteinase inhibitors [1−8]. Agents targeting the human epidermal growth factor receptor HER 2, epidermal growth factor receptor 1 (EGFR), vascular endothelial growth factor (VEGF), and cell cycle regulators are being integrated into therapeutic studies with the goal of improving therapeutic efficacy and patient outcome. Approximately 25−30% of breast cancers that overexpress HER-2 will respond positively to HER-2 targeted therapies such as Trastuzumab. However, some patients develop resistance to Trastuzumab within one year of treatment [9]. Therefore, it is important to explore new molecular targets and develop novel targeted drugs for the treatment of breast cancer patients. In addition to the HER-2 gene, several other important genes closely related to breast carcinogenesis such as BRCA1, P53, HIC-1, and TOP2A are also located on chromosome 17 [10]. HIC-1 is a tumor suppressor gene that is expressed at low levels in breast cancer and other malignancies due to epigenetic silencing [11−16]. However, the precise molecular pathways and functional mechanisms that regulate its expression are poorly understood.

Several successful molecularly targeted drugs have been developed, including Trastuzumab (Herceptin), Gefitinib (Iressa), and Bevacizumab (Avastin). 5-aza-2′deoxycytidine (5-CdR) is a non-specific demethylation drug that can reactivate tumor suppressor genes by demethylation. In 2006 and 2007, two reports from the Li and Janowski groups revealed that double-stranded small RNA (dsRNA) molecules could activate the expression of target genes by binding to the promoter region upstream of the transcription start site [17], [18]. These important findings open the door for gene therapies targeting tumor suppressor genes. They termed the dsRNAs as small activating RNA (saRNA) and the molecular event RNA activation (RNAa), which is the opposite of the classical phenomenon of RNA interference (RNAi) [19−21]. To verify the efficacy of saRNAs on hypermethylated tumor suppressors, we previously created several saRNAs targeted to the HIC-1 promoter and assessed the effect of re-expression on gastric cancer. Our study indicated that saRNA-mediated the re-expression of HIC-1 and inhibited cell proliferation, migration, and clonogenicity, and induced apoptosis. Therefore, HIC-1 is a potential target for gene therapy in gastric cancer, and saRNAs could present a novel therapeutic option for upregulating tumor suppressor genes [22].

In the present study, we assessed HIC-1 as a candidate therapeutic target and observed altered cellular functions after the re-expression of HIC-1 in breast cancer cells. Several saRNAs were used for RNA activation in combination with 5-CdR treatment in breast cancer. To clarify the molecular mechanisms and related pathways modulated by HIC-1 activation, we also screened the differentially expressed genes by whole transcriptomic microarray.

## Materials and Methods

### Tissue microarray, cell lines, and reagents

A breast cancer tissue microarray (OD-CT-RpBre01-003) was purchased from Outdo Biotech Co, Ltd. (Shanghai, China), which contains 80 breast cancer samples from patients aged 33−81 years (average 55). Ten samples had paired adjacent normal breast tissue. Fifty-five cases were invasive ductal carcinoma, six were invasive lobular carcinoma, nine mucinous carcinomas, four medullary carcinomas, three lipid-rich carcinomas, and three were intraductal carcinoma.

The breast cancer cell lines MCF-7 and MDA-MB-231 were purchased from the Cell Bank of Chinese Academy of Sciences Type Culture Collection Committee. The human normal mammary epithelial cell line MCF-10A was obtained from Shanghai Institute of Breast Cancer. DMEM medium, fetal bovine serum (FBS), and horse serum were purchased from Hyclone (Thermo Fisher Scientific, USA). 5-CdR was purchased from Sigma-Aldrich (USA), dissolved in PBS to make a 10 mM stock solution, and stored at −80°C. Complete medium was diluted to working concentrations before use. TRIzol was purchased from Invitrogen (USA). The CCK-8 kit was purchased from Dojindo (Japan). RT-PCR and MSP kits were purchased from TaKaRa (Japan). DNA extraction and bisulfate conversion kit were purchased from Qiagen (Germany). RT-PCR and MSP primers were synthesized by Shanghai Sangon Ltd.

### Ethics Statement

Written informed consent for the study was obtained from all participants. The ethics committee of Outdo Biotech Co, Ltd., Shanghai, approved the study protocol.

### Immunohistochemistry for HIC-1 staining

Tissue microarrays were de-waxed, and hydrated using alcohol. Antigen retrieval was performed using citrate buffer, and endogenous peroxide activity was blocked with a 3% hydrogen peroxide solution. Mouse anti-human HIC-1 monoclonal antibody (1∶100, ab55120, Abcam, UK) was added, followed by incubation at 37°C for 1 h, and three washes with 1× PBS for 5 min. Then EnVision two-step reagents (Dako) were then incubated at 37°C for 30 min. DAB was used for signal detection, and hematoxylin was used for nuclear staining. HIC-1 expression in the nucleus or cytoplasm was judged to be positive. Tissue microarrays were scored according to the proportion of positive cells and staining intensity. For cell proportion scoring, positive cells <10% = 0, 10−30% = 1, 31−50% = 2, and >50% _as_ 3. In staining intensity scoring, no stain = 0, pale yellow = 1, brown/yellow = 2, and dark brown = 3. The final scores were obtained by multiplying the two. Scores of 1−4 were weakly positive, and >6 designated retained expression.

### Cell culture and methylation analysis

We resuspended MCF-7 and MDA-MB-231 cells in DMEM medium containing 10% FBS, and 2×10^5^ cells were seeded at 6-well plates and incubated at 37°C with 5% CO_2_. Media containing 5, 10, 20, 40, or 80 µM 5-CdR were added, and cells were incubated for at least 24 h. At days 2, 4, and 5, cells were harvested, and genomic DNA was extracted using a QIAamp DNA Mini Kit. DNA was treated with sodium bisulfate following the specifications provided. The primer sequences for methylated HIC-1 promoter were F (5′-TCGGTTTTCGCGTTTTGTTCGT-3′), R (5′-AACCGAAAACTATAAACCCTCG-3′) with a 95 bp amplification product. The primer sequences for the unmethylated HIC-1 promoter were F (5′-TTGGGTTTGGTTTTTGTGTTTTG-3′), and R (5′-CACCCTAACACCACCCTAAC-3′), with a 181 bp amplification product. Reactions were hot-started at 95°C for 30 s, followed by 40 cycles of 94°C for 30 s, 62°C for 30 s, and 72°C for 30 s, followed by 72°C for 10 min. The PCR products were analyzed on 1.5% agarose gels, stained with GelRed, and visualized by UV illumination.

### mRNA analysis

Total RNA was isolated using TRIzol and reverse transcribed using PrimeScript® RT master mix random primers. RT-PCR was performed using EmeraldAmp® PCR Master Mix. The primer sequences for the RT-PCR reactions were follows: HIC-1 F (5′-GTCGTGCGACAAGAGCTACAA-3′), R (5′-CGTTGCTGTGCGAACTTGC-3′), which amplify a 282 bp product. GAPDH: F (5′-CCTGCACCACCAACTGCTTA-3′), R (5′-AGGCCATGCCAGTGAGCTT-3′), giving a 178 bp product. Reactions were hot-started at 98°C for 3 min, followed by 35 cycles of 98°C for 30 s, 60/55°C (HIC-1/GAPDH) for 30 s, and 72°C for 30 s, followed by 72°C for 10 min. The PCR products were analyzed on 1.5% agarose gels, stained with GelRed, and visualized by UV illumination.

### Cell proliferation assay (CCK-8)

Cell Counting Kit 8 was used to assess cell proliferation. Briefly, control and treated cancer cells (2×10^3^ cells/well) were seeded onto 96-well plates. At the specified time points, 10 µl of CCK-8 solution was added to each well of the plate, and then incubated for 2 h. Cell viability was determined by measuring the OD at 450 nm using a microplate reader.

### Apoptosis assay

Experimental cells were treated with 5-CdR or medium daily for 5 days. Then, cells were collected and washed. Annexin V-FITC Apoptosis Detection Kit (BD Pharmingen, San Jose, CA, USA) was assayed according to the manufacturer's instructions. Briefly, cells were washed with PBS and resuspended in 1× binding buffer at a concentration of 1×10^6^ cells/ml. Next, 5 µl of FITC Annexin V and 5 µl of PI were added to 100 µl of the cell suspension, and incubated for 15 min in the dark. After incubation, 400 µl 1× binding buffer was added, and apoptotic cells were analyzed using a FACScan flow cytometer (Beckman Instruments, Fullerton, CA, USA).

### saRNA design and transfection

All dsRNAs targeting the region upstream of the transcriptional start site of human HIC-1 were designed based on the rational design rules [17], [23]. Four dsRNAs targeting the −3000 bp upstream region from the transcription start site (TSS) of the HIC-1 gene were designed and synthesized (Shanghai Genepharma Company, China). dsRNA targeting −29 (dsHIC1-29): F- CAGAUAAGAGUGUGCGGAATT, R- UUCCGCACACUCUUAUCUGTT. dsRNA targeting −1873 (dsHIC1-1873): F- GGGAUCUGACUCUAUCAAATT, R- UUUGAUAGAGUCAGAUCCCTT. dsRNA targeting −2873 (dsHIC1-2873): F- AGAUGGAGGAAGGGUCUAATT, R- UUAGACCCUUCCUCCAUCUTT. dsRNA targeting −2998 (dsHIC1-2998): F- CGGUUUCCUGGAGAAGUUATT, R- UAACUUCUCCAGGAAACCGTT. dsRNA for negative control (dscontrol): F- ACGUGACACGUUCGGAGAATT, R- UUCUCCGAACGUGUCACGUTT. MCF-7 and MDA-MB-231 cells were trypsinized, diluted in growth medium without antibiotics, and seeded in 6-well plates (4.0×10^5^ cells/well). dsRNAs were transfected at a concentration of 50 nM/L using Lipofectamine 2000 (Life Technologies, Carlsbad, CA) according to the reverse transfection protocol provided with the product. The cells were harvested 3−5 days after transfection for further analysis.

### Gene chip experiment

A functional small RNA fragment of dsHIC1-2998 was transfected into MCF-7 and MDA-MB-231 cells. Cells were harvested 96 h after transfection and washed twice with PBS. Total RNA was extracted using TRIzol Reagent (Cat#15596-018, Life Technologies) following the manufacturer's instructions, and an RNA integrity number (RIN) was assigned to assess RNA integration using an Agilent Bioanalyzer 2100 (Agilent Technologies, Santa Clara, CA, US). Qualified total RNA was further purified using a RNeasy micro kit (Cat#74004, Qiagen, GmBH, Germany) and RNase-Free DNase Set (Cat# 79254, Qiagen). Total RNA was amplified and labeled by Low Input Quick Amp Labeling Kit, One-Color (Cat# 5190-2305, Agilent), following the manufacturer's instructions. Labeled cRNAs were purified using an RNeasy mini kit (Cat#74106, Qiagen). Each slide (Agilent human whole genome 4*44 chip) was hybridized with 1.65 µg Cy3-labeled cRNA using a Gene Expression Hybridization Kit (Cat#5188-5242, Agilent) in a hybridization oven (Cat# G2545A, Agilent), according to the manufacturer's instructions. After a 17 h hybridization, slides were washed in staining dishes (Cat#121, Thermo Shandon, Waltham, MA, US) with Gene Expression Wash Buffer Kit (Cat#5188-5327, Agilent), following the manufacturer's instructions. Slides were scanned using an Agilent Microarray Scanner (Cat#G2565CA, Agilent) with default settings: dye channels: green, scan resolution = 5 µm, PMT 100%, 10%, 16 bit. Data were extracted using Feature Extraction software 10.7 (Agilent). Raw data were normalized using the Quantile algorithm, Gene Spring Software 11.0 (Agilent). Welch's *t*-tests and the Significance Analysis of Microarray (SAM) tests were used to identify genes that were differentially expressed in the trial subjects of each category, and *P*<0.01 and fold-changes ≥2 or ≤−2 were used as the filters for screening genes. A two-way clustering algorithm was used to analyze the distribution of samples and genes. Microarray data were deposited in the Gene Expression Omnibus at http://www.ncbi.nlm.nih.gov/geo (accession ID:GSE42024).

### Activating efficacy of HIC-1 by quantitative RT-PCR

Total RNA was extracted using TRIzol reagent (Life Technologies). Real-time PCR amplification of the cDNA was performed in a reaction mixture with a final volume of 20 µl containing 10 µl of SYBR Green PCR Master Mix (Applied Biosystems, USA), 1 µl of 5 mM/L each paired primer specific to target gene, and 1 µl of cDNA. The primers used for real time PCR were: HIC-1 forward, 5′- TAAATCGGGAGAGTGTGCTGGGC-3′, and reverse, 5′- GTGCGCTGGTTGTTGAGCTGC-3; GAPDH forward, 5′- GGACCTGACCTGCCGTCTAG-3′, and reverse, 5′- GTAGCCCAGGATGCCCTTGA-3′.

### Statistical analysis

Statistical analyses were performed using the software package SPSS 15.0. The measurement data were analyzed by *t*-test, and numeration data were analyzed using the fourfold table χ2 test or Fisher's exact test. Differences were considered significant at *P*<0.05.

## Results

### HIC-1 protein expression in breast cancer tissue arrays

**Figure 1:**
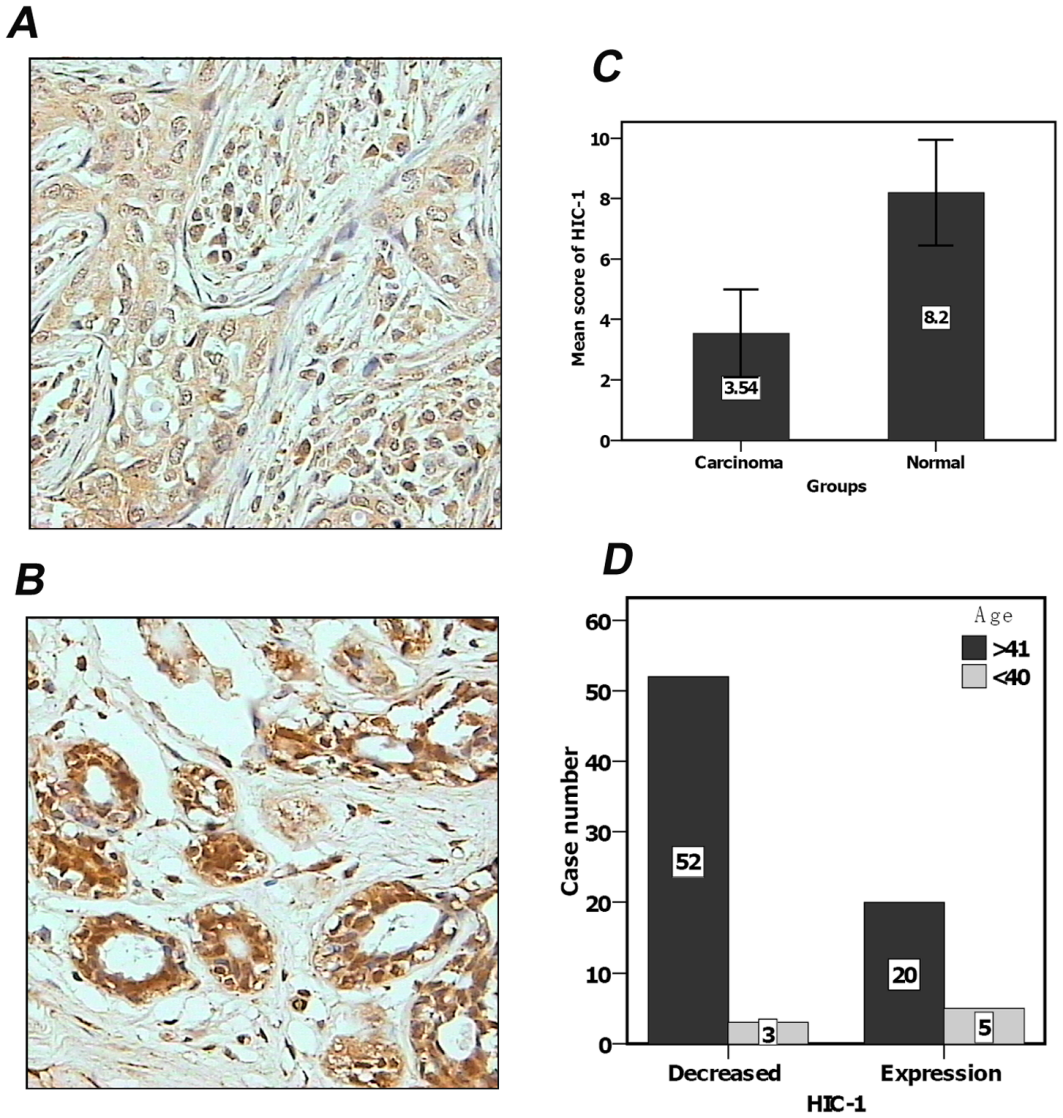
Immunohistochemical staining and scoring analysis of HIC-1 in breast cancer tissues and adjacent normal breast tissue. A. HIC-1 protein expression was decreased in breast cancer tissue (400×). B. The expression of HIC-1 protein was strong in normal breast epithelial tissue (400×). C. Bar chart of immunohistochemical scores. Normal breast epithelial tissue scored 8.2±1.75 points, whereas the average score of breast cancer was 3.54±1.46. There were significant differences between the two groups (*P*<0.001). D. The expression of HIC-1 was higher in younger patients compared with older patients (*P*<0.05).

Staining was judged to be positive when yellow or brown granules appeared in the nucleus or cytoplasm of cells. Based on HIC-1 immunohistochemistry, a final score<4 was considered to be decreased expression (Figure 1A), and a final score>6 as retained expression. HIC-1 protein expression was strongly positive in normal breast epithelial tissue (Figure 1B). The average score of 10 samples of paired normal breast epithelial was 8.2. The expression of HIC-1 protein was reduced significantly in breast cancer tissue. In 80 breast cancer samples, 55 (62.5%) exhibited low-expression, and 25 positive expression. The average score of HIC-1 expression was 3.54±1.46 in breast cancer. There was a significant difference in HIC-1 expression between cancerous and normal breast tissue (8.2±1.75, *P*<0.001, Figure 1C). Of the breast cancer samples, 55 were primary breast cancer without metastasis, and 25 exhibited metastasis in the axillary lymph nodes. The correlation between HIC-1 protein expression and clinicopathological parameters is shown in Table 1. There was no significant relationship between HIC-1 protein expression, tumor location, lymph node metastasis, and histological sub-type. However, HIC-1 protein expression was significant correlated with patient age (Figure 1D). The expression level of HIC-1 was higher in younger patients than in older patients (*P*<0.05).

**Table 1:** HIC-1 protein expression and clinical pathology parameters.

Parameter	Decreased expression	Retained expression	*P* value
**Age**			
<40	3	5	0.044
>41	52	20	
**Location**			
Left breast	32	11	0.238
Right breast	23	14	
**Histology**			
Invasive ductal carcinoma	38	17	0.549
Invasive lobular carcinoma	3	3	
Others	14	5	
**Lymph node metastasis**			
Negative	37	18	0.672
Positive	18	7	

### 5-CdR treatment partially restored HIC-1 expression in breast cancer cell lines

**Figure 2:**
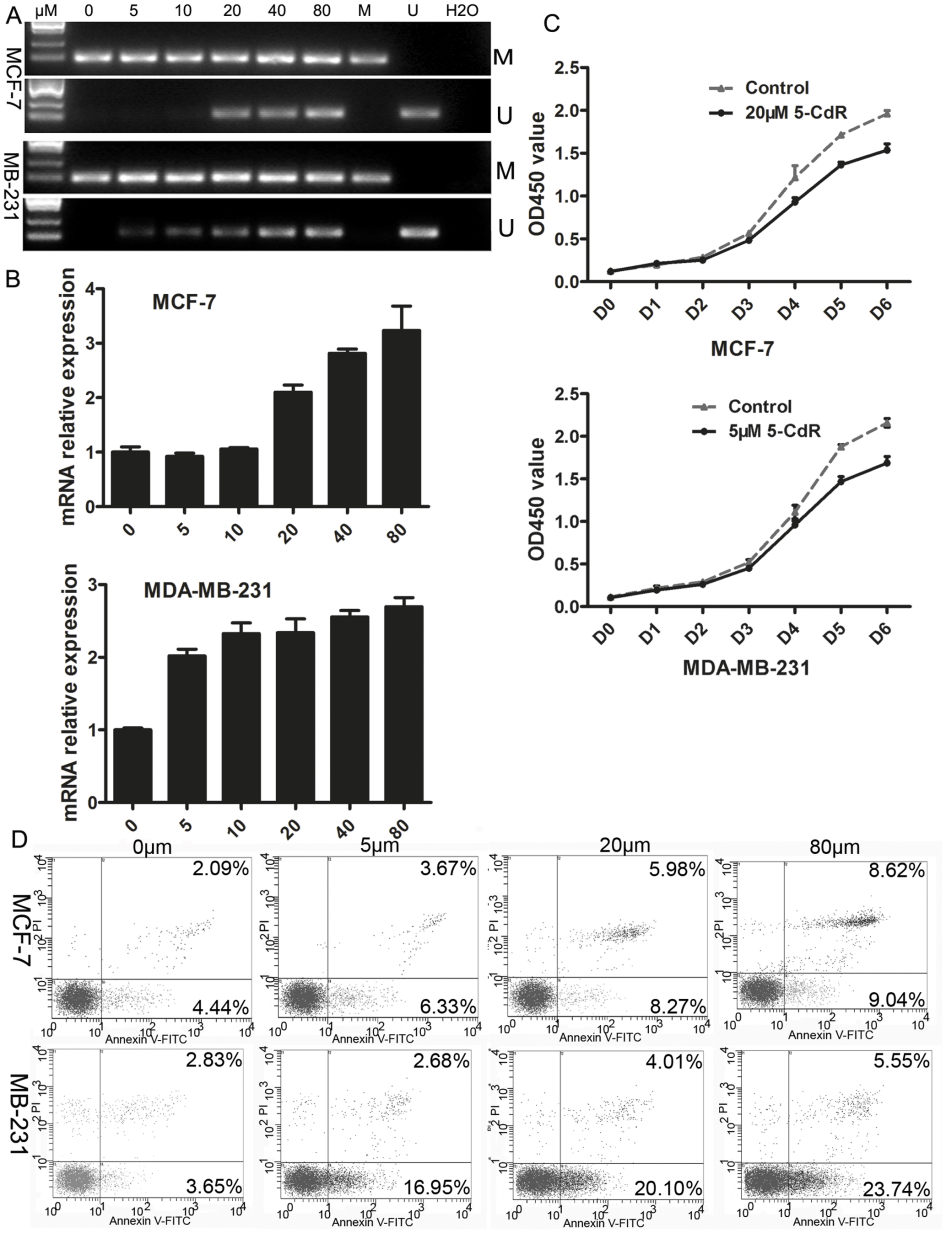
The effects of different concentrations of 5-CdR on HIC-1 gene expression. A. Unmethylated bands appeared gradually with increasing drug concentrations. However, the methylation of the HIC-1 promoter region could not be eliminated completely (upper, MCF-7; lower, MDA-MB-231). M: methylated band (95 bp), U: unmethylated band (181 bp). B. After 5-CdR treatment for 5 days, HIC-1 expression was increased gradually in a concentration-dependent manner. C. Cell proliferation curves of MCF-7 (upper) and MBA-MB-231 cells (lower) after treatment with 20 and 5 µM 5-CdR respectively. Cell growth was inhibited significantly in both cancer cell lines from day 3 of 5-CdR treatment. D. The total apoptosis in MCF-7 cells (top) was increased significantly, compared with the control group after treatment with 5, 20, and 80 µM 5-CdR for 5 days (10±1.44%, 14.25±0.82%, and 17.66±1.53%, respectively, vs. 6.53%±1.38%, *P*<0.05). The total percentage of apoptotic MDA-MB-231 cells (lower) was also increased significantly compared with control (19.63±1.58%, 24.11±1.03%, and 29.29±1.14%, respectively, vs. 6.48±1.37%, *P*<0.05).

MDA-MB-231 and MCF-7 cells were treated with 0, 5, 10, 20, 40, or 80 µM 5-CdR for 5 days. Although unmethylated bands were detected with increasing drug concentrations, the methylated HIC-1 promoter region could not be eliminated completely in either cell line (Figure 2A). This suggests that 5-CdR partially reversed the methylation of the HIC-1 promoter. After 5-CdR treatment for 5 days, HIC-1 expression increased gradually in a dose-dependent manner (Figure 2B), suggesting that (in addition to de-methylation) the expression of the HIC-1 gene was restored gradually. We analyzed the proliferation curves of MCF-7 and MDA-MB-231 cells treated with 20 and 5 µM 5-CdR, respectively, and observed that cell growth was inhibited significantly in both cancer cells from day 3 of 5-CdR treatment (Figure 2C, *P*<0.05).

### 5-CdR treatment induced apoptosis in breast cancer cell lines

Apoptosis was assessed in MCF-7 and MDA-MB-231 cells after treatment with 5, 20 or 80 µM 5-CdR for 5 days. The total apoptosis was increased significantly in MCF-7 cells compared with control (10±1.44%, 14.25±0.82%, and 17.66±1.53% vs. 6.53±1.38%, *P*<0.05). Similarly, the total apoptosis was also increased in MDA-MB-231 cells compared with control (19.63±1.58%, 24.11±1.03% and 29.29±1.14% vs. 6.48±1.37%, *P*<0.05) (Figure 2D). This suggests that restoring HIC-1 expression induced apoptosis in breast cancer cells.

### saRNA restored HIC-1 expression in breast cancer cells

**Figure 3:**
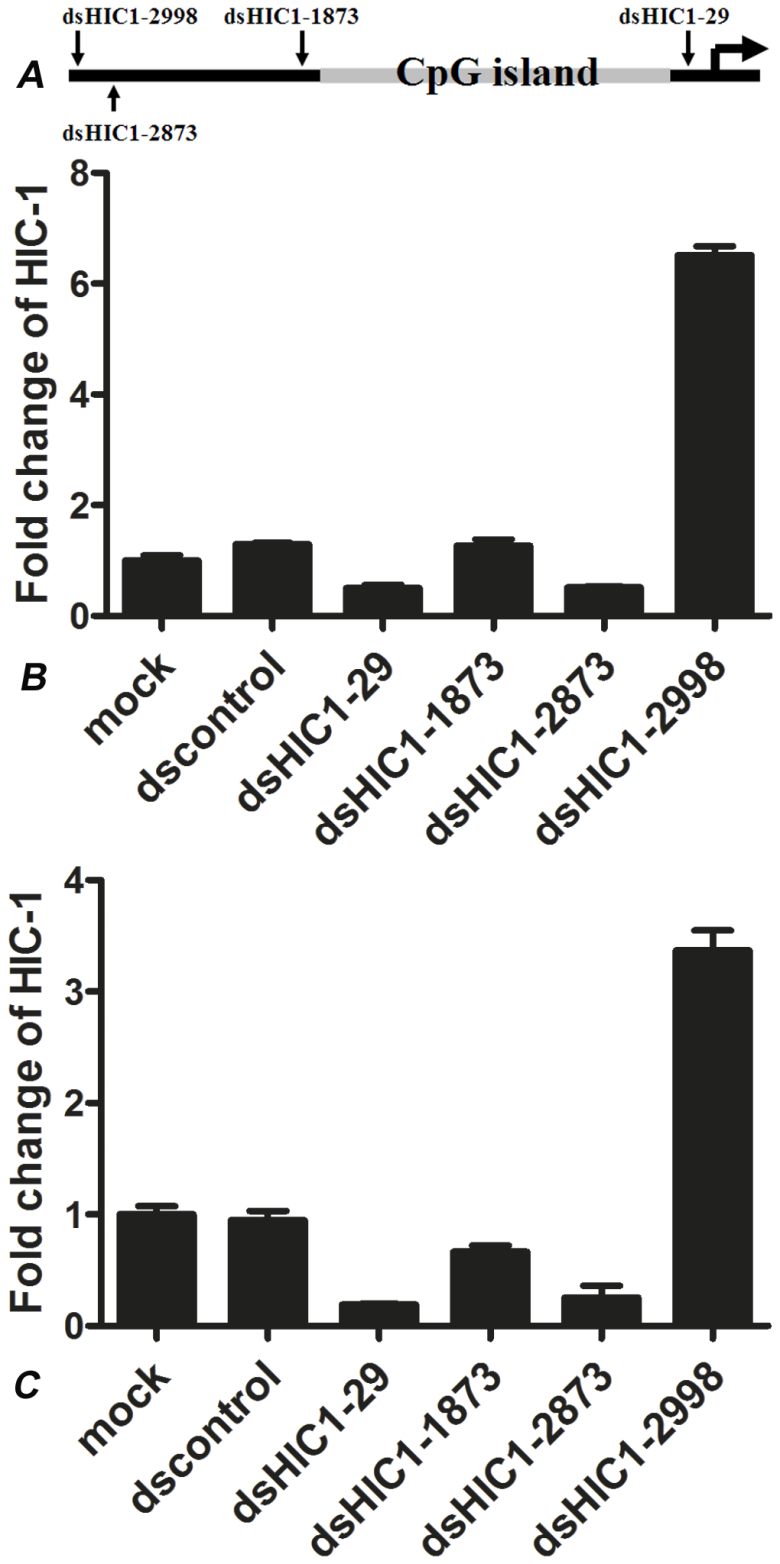
Restoration of HIC-1 expression by saRNA (dsHIC1-2998) in breast cancer cells. A. The binding sites of four saRNAs targeting the HIC-1 promoter. All the dsRNAs were separate from CpG islands in the HIC-1 promoter. B. In MCF-7 cells, HIC-1 mRNA levels were upregulated 6.52-fold by dsHIC1-2998 transfection, compared with mock transfection (*P*<0.01). In MDA-MB-231 cells, HIC-1 mRNA levels were 3.37-fold upregulated by dsHIC1-2998 compared with mock (*P*<0.01).

Four candidate saRNAs were synthesized that targeted the promoter regions of −29 (dsHIC1-29), −1873 (dsHIC1-1873), −2873 (dsHIC1-2873), and −2998 (dsHIC1-2998). As shown in Figure 3A, all the binding sites for saRNAs were distant from the CpG islands of the HIC-1 promoter. Four saRNAs were transfected into MCF-7 and MDA-MB-231 cells, and HIC-1 mRNA expression was assessed by quantitative RT-PCR 4 days after transfection. In MCF-7 cells, HIC-1 mRNA levels were upregulated 6.52-fold by dsHIC1-2998 transfection compared with mock (*P*<0.01). In contrast, dsHIC1-29, dsHIC1-1873, and dsHIC1-2873 did not affect HIC-1 mRNA levels significantly (Figure 3B). Similarly in MDA-MB-231 cells, HIC-1 mRNA levels were upregulated 3.37-fold by dsHIC1-2998 compared with mock (*P*<0.01). In contrast, dsHIC1-29, dsHIC1-1873, and dsHIC1-2873 did not alter HIC-1 mRNA levels significantly (Figure 3C). Therefore, dsHIC1-2998 was selected as the effective saRNA for additional studies.

**Figure 4:**
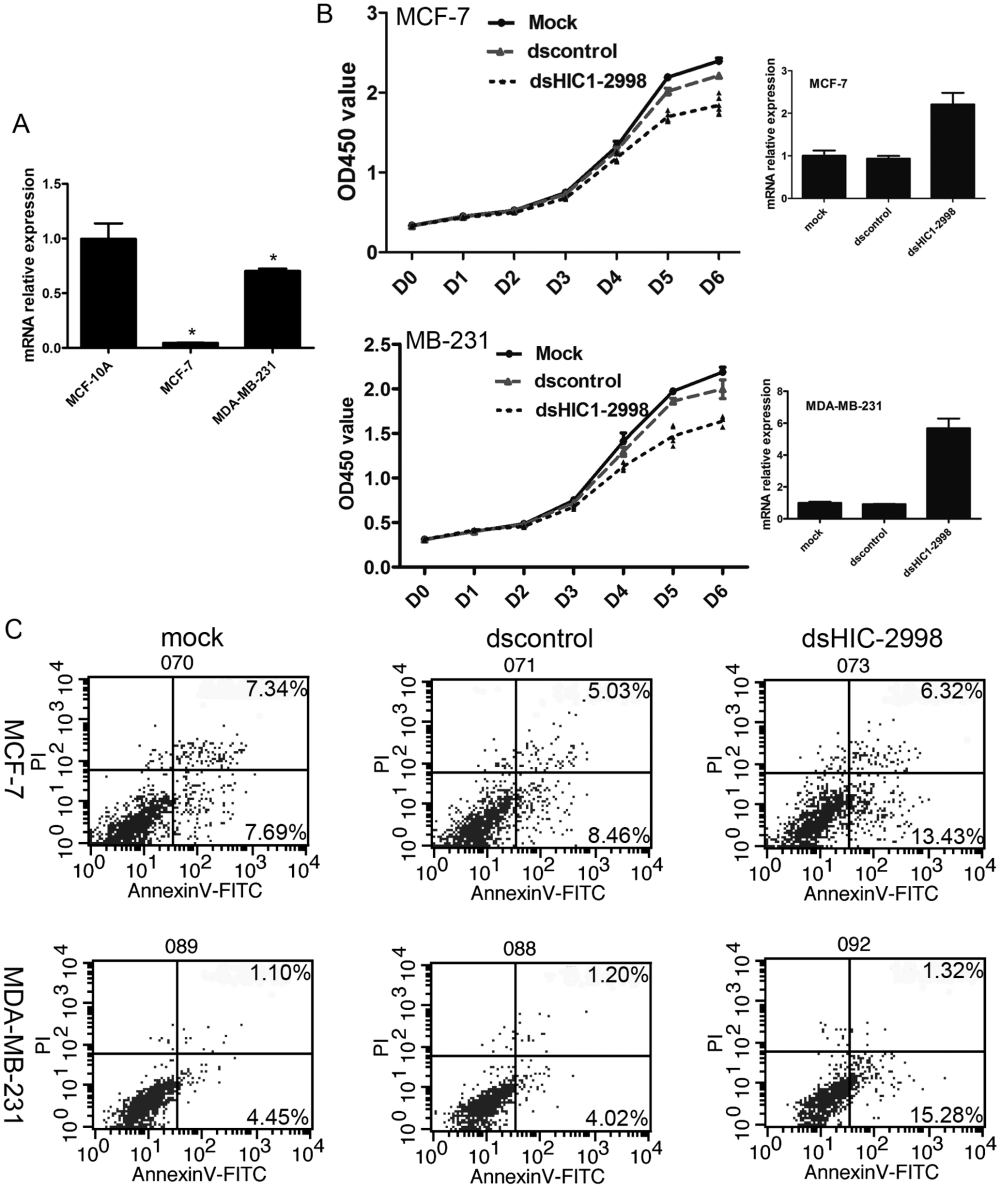
Upregulation of HIC-1 suppresses cell growth and induces apoptosis after dsHIC1-2998 transfection. A. The basal expression levels of HIC-1 in MCF-7 and MDA-MB-231 cancer cells and MCF-10A normal mammary epithelial cells. The expression of HIC-1 mRNA in the breast cancer cell lines MCF-7 and MDA-MB-231 was significantly lower than in the normal mammary epithelial cell line MCF-10A (*P*<0.05). B. Reactivation of HIC-1 using the RNAa dsHIC1-2998 inhibited breast cancer cell viability. MCF-7 cells (upper) and MDA-MB-231 (lower) cells were transfected with 50 nmol/L dsRNA, and cell proliferation was assayed at each time point. Data are plotted as mean ± SD. C. Upregulation of HIC-1 promoted total apoptosis in MCF-7 (upper) and MDA-MB-231 (lower) cells after dsHIC1-2998 transfection.

Figure 4A revealed that the expression of HIC-1 mRNA was significantly lower in breast cancer cell lines MCF-7 and MDA-MB-231 than in the normal mammary epithelial cell line MCF-10A. Relative to a value of 1 for the mRNA expression in MCF-10A cells, the relative HIC-1 expression was 0.045 and 0.703 in MCF-7 and MDA-MB-231, respectively. dsHIC1-2998 (50 nmol/L) was then transfected into MCF-7 and MDA-MB-231 breast cancer cells, and HIC-1 mRNA expression was evaluated using real-time PCR four days after saRNA transfection. In MCF-7 cells, HIC-1 mRNA levels were upregulated 2.2 fold compared with control (*P*<0.01). In MDA-MB-231 cells, HIC-1 levels were upregulated 5.7-fold compared with control (*P*<0.01) (Figure 4B). We analyzed cell proliferation curves based on dsHIC1-2998 transfection, and assayed OD450 values serially for 6 days. From days 3−6, OD450 values were reduced significantly in the dsHIC1-2998-tranefected MCF-7 and MDA-MB-231 cells compared with controls (*P*<0.05) (Figure 4C). Seventy-two hours after dsHIC1-2998 transfection, cells were harvested and stained with annexin-V-FITC and PI, and apoptosis was analyzed using flow cytometry. In MCF-7 cells, the number of total apoptotic dsHIC1-2998-transfected cells was increased significantly compared with mock (19.75% *vs.* 15.03%, P<0.05). Similarly, the percentage of total apoptotic dsHIC1-2998-transfected MDA-MB-231 cells was increased significantly compared with mock (16.60% *vs.*5.55%, P<0.05, Figure 4D).

### Identification of differentially expression genes after the reactivation of HIC-1

**Figure 5:**
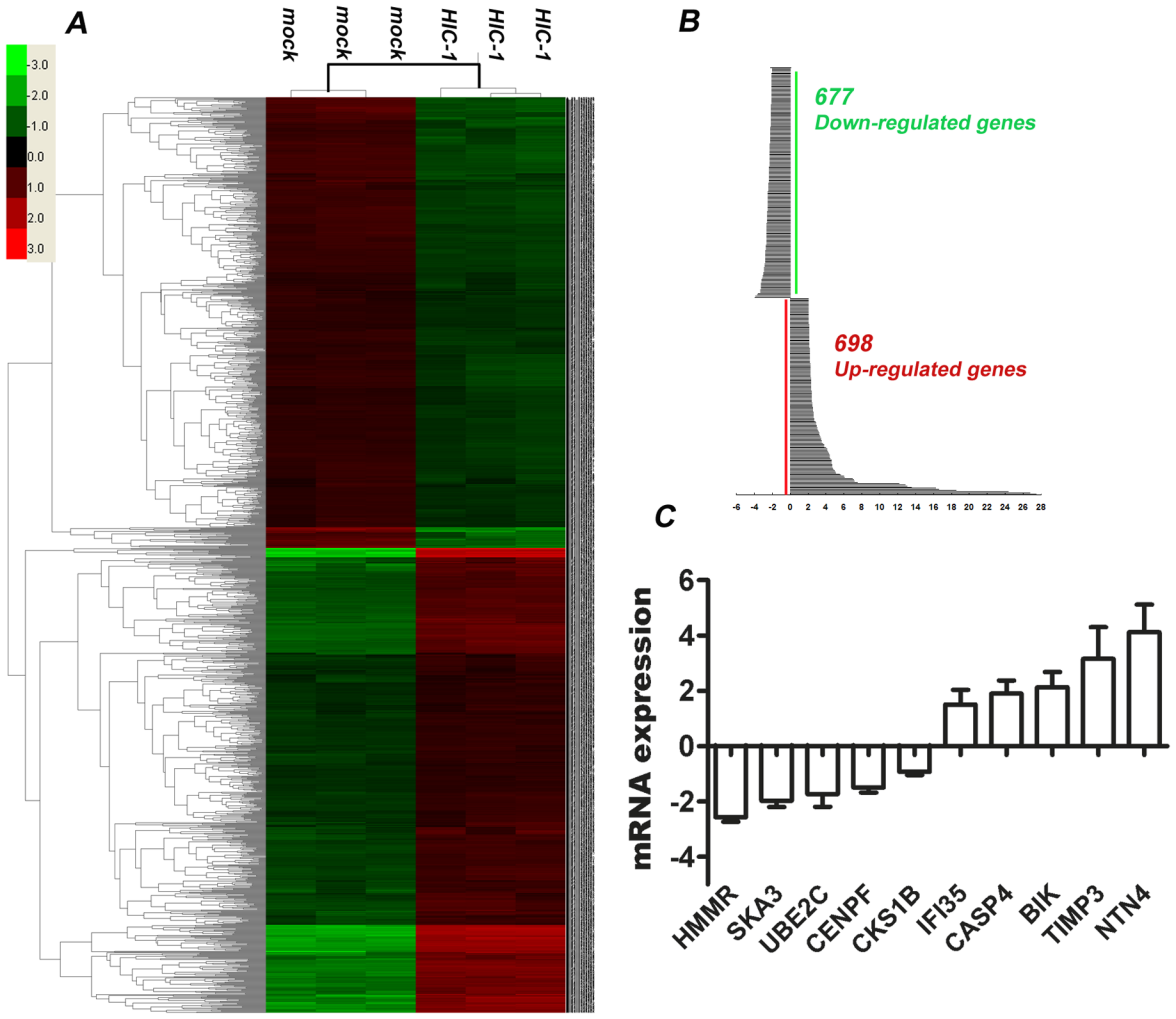
Gene expression profiles of whole transcriptome microarrays. A. Two-way hierarchical clustering heatmap of differentially expressed genes between HIC-1 activated MCF-7 cells and control. Gene expression was significantly different between the two groups. B. Bar chart of up- and downregulated genes based on HIC-1 reactivation in MCF-7 cancer cells. C. mRNA verification of 10 selected genes by quantitative RT-PCR on the same six samples used in the microarray study. Five genes were upregulated (TIMP3, NTN4, BIK, CASP4, and IFI35), and five were downregulated (SKA3, HMMR, CENPF, CKS1B, and UBE2C), which is consistent with the results of the microarray assay.

We assayed the differentially expressed genes in cancer cells using whole transcriptomic microarrays after restoring HIC-1 expression using dsHIC1-2998 saRNA. Microarray data can be obtained from the Gene Expression Omnibus at http://www.ncbi.nlm.nih.gov/geo (GSE42024). Two standards of statistical value (*P*<0.01) and fold-change (≥2 or ≤−2) were used as the filtering criteria. Six samples from the HIC-1 reactivation and control groups were analyzed by two-way clustering. A total of 1375 (698 upregulated and 677 downregulated) genes were identified in saRNA treated MCF-7 cells (Figure 5A, 5B). To understand the differentially expressed genes, we present representative genes and their fold-change in Tables 2 and 3. The upregulated genes include those involved in the immune network, antigen processing and presentation, and developmental growth. In contrast, the downregulated genes play roles in processes including cancer, the cell cycle, chromosome segregation, and cell division. The differentially expressed genes in MDA-MB-231 cells are presented in Figure S1, Tables 4 and Tables 5. To confirm the reliability of the microarray, we assessed the mRNA expression of 10 selected genes by quantitative RT-PCR on the original samples used in the microarray: five that were upregulated (TIMP3, NTN4, BIK, CASP4, and IFI35), and five that were downregulated (SKA3, HMMR, CENPF, CKS1B, and UBE2C). The changes in expression of all selected genes were verified in the dsHIC-2998 transfection group compared with control by quantitative RT-PCR (Figure 5C, *P*<0.05).

**Table 2:** Up-regulated genes upon HIC-1 re-expression in MCF-7*.

Gene symbol	Gene description	Genbank ID	Fold-change
DEFB4A	defensin, beta 4A	NM_004942	27.435
S100A7	S100 calcium binding protein A7	NM_002963	26.783
S100A8	S100 calcium binding protein A8	NM_002964	18.481
DEFB1	defensin, beta 1	NM_005218	16.587
S100A9	S100 calcium binding protein A9	NM_002965	16.199
S100A12	S100 calcium binding protein A12	NM_005621	13.528
CFB	complement factor B	NM_001710	13.032
PLA2G2A	phospholipase A2, group IIA	NM_000300	9.401
INHBA	inhibin, beta A	NM_002192	8.800
FBXO32	F-box protein 32	NM_058229	8.258
GSTA5	glutathione S-transferase alpha 5	NM_153699	7.987
GPX2	glutathione peroxidase 2	NM_002083	7.134
GSTA2	glutathione S-transferase alpha 2	NM_000846	6.125
ALDH1A3	aldehyde dehydrogenase 1 family, member A3	NM_000693	6.108
TIMP3	TIMP metallopeptidase inhibitor 3	NM_000362	5.611
IFNGR1	interferon gamma receptor 1	NM_000416	5.493
MALL	mal, T-cell differentiation protein-like	NM_005434	5.179
TNFAIP2	tumor necrosis factor, alpha-induced protein 2	NM_006291	5.010
NTN4	netrin 4	NM_021229	4.563
CARD6	caspase recruitment domain family, member 6	NM_032587	4.335
CAPN13	calpain 13	NM_144575	3.591
ALDH3B1	aldehyde dehydrogenase 3 family, member B1	NM_000694	3.457
HSPB8	heat shock 22 kDa protein 8	NM_014365	3.409
GLRX	glutaredoxin (thioltransferase)	NM_002064	3.063
SOD2	superoxide dismutase 2, mitochondrial	NM_001024465	2.925
BIK	BCL2-interacting killer (apoptosis-inducing)	NM_001197	2.779
CASP4	caspase 4, apoptosis-related cysteine peptidase	NM_033306	2.547
HOXA5	homeobox A5	NM_019102	2.528
MUC20	mucin 20, cell surface associated	NM_152673	2.457
GDF15	growth differentiation factor 15	NM_004864	2.449
ANXA3	annexin A3	NM_005139	2.399
HOXB2	homeobox B2	NM_002145	2.382
PLA2G10	phospholipase A2, group X	NM_003561	2.300
KLF5	Kruppel-like factor 5	NM_001730	2.194
IFI35	interferon-induced protein 35	NM_005533	2.172
TMPRSS13	transmembrane protease, serine 13	NM_001206790	2.161
LAMC1	laminin, gamma 1	NM_002293	2.146
IFI44	interferon-induced protein 44	NM_006417	2.122
KLF7	Kruppel-like factor 7	NM_003709	2.111
PRR15	proline rich 15	NM_175887	2.073
LTBR	lymphotoxin beta receptor	NM_002342	2.038

* with filters of *P*<0.01 and fold-changes ≥2.

**Table 3:** Down-regulated genes upon HIC-1 re-expression in MCF-7*.

Gene symbol	Gene description	Genbank ID	Fold-change
KREMEN2	kringle containing transmembrane protein 2	NM_172229	−5.753
ZNF695	zinc finger protein 695	NM_020394	−4.125
TNNT1	troponin T type 1 (skeletal, slow)	NM_01126132	−3.892
RHOH	ras homolog gene family, member H	NM_004310	−3.722
TFF3	trefoil factor 3 (intestinal)	NM_003226	−3.666
CRLF1	cytokine receptor-like factor 1	NM_004750	−3.551
CENPA	centromere protein A	NM_001809	−3.329
PTTG1	pituitary tumor-transforming 1	NM_004219	−3.303
E2F7	E2F transcription factor 7	NM_203394	−3.265
CASC5	cancer susceptibility candidate 5	NM_170589	−3.245
YBX2	Y box binding protein 2	NM_015982	−3.210
PTTG2	pituitary tumor-transforming 2	NM_006607	−3.192
AURKB	aurora kinase B	NM_004217	−3.185
PBK	PDZ binding kinase	NM_018492	−3.149
RASIP1	Ras interacting protein 1	NM_017805	−3.129
MGP	matrix Gla protein	NM_000900	−3.097
CDCA7	cell division cycle associated 7	NM_031942	−3.012
NUSAP1	nucleolar and spindle associated protein 1	NM_016359	−2.963
HMMR	hyaluronan-mediated motility receptor	NM_012484	−2.959
TMEM121	transmembrane protein 121	NM_025268	−2.949
SKP2	S-phase kinase-associated protein 2	NM_032637	−2.921
HMGB2	high mobility group box 2	NM_002129	−2.918
CCNA2	cyclin A2	NM_001237	−2.904
CDC25C	cell division cycle 25 homolog C	NM_001790	−2.887
BMP7	bone morphogenetic protein 7	NM_001719	−2.849
CENPW	centromere protein W	NM_01012507	−2.810
MKI67	antigen identified by antibody Ki-67	NM_002417	−2.798
RET	ret proto-oncogene	NM_020975	−2.776
CCNB2	cyclin B2	NM_004701	−2.754
SKA3	spindle and kinetochore associated complex subunit 3	BC013418	−2.720
TOP2A	topoisomerase (DNA) II alpha	NM_001067	−2.717
PLK4	polo-like kinase 4	NM_014264	−2.709
OIP5	Opa interacting protein 5	NM_007280	−2.707
ANP32E	Acidic nuclear phosphoprotein 32 family, member E	NM_030920	−2.669
CENPF	centromere protein F	NM_016343	−2.632
RAB31	RAB31, member RAS oncogene family	NM_006868	−2.556
PLK1	polo-like kinase 1	NM_005030	−2.472
CKS1B	CDC28 protein kinase regulatory subunit 1B	NM_001826	−2.333
UBE2C	ubiquitin-conjugating enzyme E2	NM_181803	−2.277
CENPE	centromere protein E	NM_001813	−2.268
E2F2	E2F transcription factor 2	NM_004091	−2.246

* with filters of *P*<0.01 and fold-changes ≤−2.

**Table 4:** Up-regulated genes upon HIC-1 reactivation in MDA-MB-231.

Gene symbol	Gene description	Genbank ID	Fold-change
DEFB4A	defensin, beta 4A	NM_004942	27.435
S100A7	S100 calcium binding protein A7	NM_002963	26.783
S100A8	S100 calcium binding protein A8	NM_002964	18.481
DEFB1	defensin, beta 1	NM_005218	16.587
S100A9	S100 calcium binding protein A9	NM_002965	16.199
S100A12	S100 calcium binding protein A12	NM_005621	13.528
CFB	complement factor B	NM_001710	13.032
PLA2G2A	phospholipase A2, group IIA	NM_000300	9.401
INHBA	inhibin, beta A	NM_002192	8.800
FBXO32	F-box protein 32	NM_058229	8.258
GSTA5	glutathione S-transferase alpha 5	NM_153699	7.987
GPX2	glutathione peroxidase 2	NM_002083	7.134
GSTA2	glutathione S-transferase alpha 2	NM_000846	6.125
ALDH1A3	aldehyde dehydrogenase 1 family, member A3	NM_000693	6.108
TIMP3	TIMP metallopeptidase inhibitor 3	NM_000362	5.611
IFNGR1	interferon gamma receptor 1	NM_000416	5.493
MALL	mal, T-cell differentiation protein-like	NM_005434	5.179
TNFAIP2	tumor necrosis factor, alpha-induced protein 2	NM_006291	5.010
NTN4	netrin 4	NM_021229	4.563
CARD6	caspase recruitment domain family, member 6	NM_032587	4.335
CAPN13	calpain 13	NM_144575	3.591
ALDH3B1	aldehyde dehydrogenase 3 family, member B1	NM_000694	3.457
HSPB8	heat shock 22 kDa protein 8	NM_014365	3.409
GLRX	glutaredoxin (thioltransferase)	NM_002064	3.063
SOD2	superoxide dismutase 2, mitochondrial	NM_001024465	2.925
BIK	BCL2-interacting killer (apoptosis-inducing)	NM_001197	2.779
CASP4	caspase 4, apoptosis-related cysteine peptidase	NM_033306	2.547
HOXA5	homeobox A5	NM_019102	2.528
MUC20	mucin 20, cell surface associated	NM_152673	2.457
GDF15	growth differentiation factor 15	NM_004864	2.449
ANXA3	annexin A3	NM_005139	2.399
HOXB2	homeobox B2	NM_002145	2.382
PLA2G10	phospholipase A2, group X	NM_003561	2.300
KLF5	Kruppel-like factor 5	NM_001730	2.194
IFI35	interferon-induced protein 35	NM_005533	2.172
TMPRSS13	transmembrane protease, serine 13	NM_001206790	2.161
LAMC1	laminin, gamma 1	NM_002293	2.146
IFI44	interferon-induced protein 44	NM_006417	2.122
KLF7	Kruppel-like factor 7	NM_003709	2.111
PRR15	proline rich 15	NM_175887	2.073
LTBR	lymphotoxin beta receptor	NM_002342	2.038

**Table 5:** Down-regulated genes upon HIC-1 reactivation in MDA-MB-231.

Gene symbol	Gene description	Genbank ID	Fold-change
FAM71E1	family with sequence similarity 71	NM_138411	−3.859
HHLA3	HERV-H LTR-associating 3	NM_001036645	−3.659
CORO2B	coronin, actin binding protein, 2B	NM_006091	−2.207
AGBL2	ATP/GTP binding protein-like 2	NM_024783	−2.201
HIST1H4K	histone cluster 1, H4k	NM_003541	−2.116
PHACTR2	phosphatase and actin regulator 2	NM_001100164	−2.062
TMEM150A	transmembrane protein 150A	NM_001031738	−2.018
TCEA3	transcription elongation factor A	NM_003196	−1.965
HIST1H2AJ	histone cluster 1, H2aj	NM_021066	−1.900
FBXO32	F-box protein 32 (FBXO32)	NM_058229	−1.854
SPRR2E	small proline-rich protein 2E	NM_001024209	−1.819
ADAMTSL3	ADAMTS-like 3	NM_207517	−1.814
PIK3IP1	phosphoinositide-3-kinase interacting protein 1	NM_052880	−1.802
ATF3	activating transcription factor 3	NM_001040619	−1.782
WFDC2	WAP four-disulfide core domain 2	NM_006103	−1.767
S100A2	S100 calcium binding protein A2	NM_005978	−1.766
HIST1H4D	histone cluster 1, H4d	NM_003539	−1.756
ANXA8L2	annexin A8-like 2	NM_001630	−1.755
PRR15	proline rich 15	NM_175887	−1.754
PDGFA	platelet-derived growth factor alpha polypeptide	NM_002607	−1.745
EDN2	endothelin 2	NM_001956	−1.725
GPR87	G protein-coupled receptor 87	NM_023915	−1.716
HSPA13	heat shock protein 70 kDa family, member 13	NM_006948	−1.713
ITGBL1	integrin, beta-like 1	NM_004791	−1.691
MED28	mediator complex subunit 28	NM_025205	−1.689
COL4A6	collagen, type IV, alpha 6	NM_033641	−1.667
GDF15	growth differentiation factor 15	NM_004864	−1.658
CTSS	cathepsin S	NM_004079	−1.623
BMP10	bone morphogenetic protein 10	NM_014482	−1.597
HIST2H2BE	histone cluster 2, H2be	NM_003528	−1.572
PF4	platelet factor 4	NM_002619	−1.572
CD109	CD109 molecule	NM_133493	−1.562
CTSL2	cathepsin L2	NM_001333	−1.553
THBS1	thrombospondin 1	NM_003246	−1.552
CD3E	CD3e molecule	NM_000733	−1.550
RDH16	retinol dehydrogenase 16	NM_003708	−1.546
CADM1	cell adhesion molecule 1	NM_014333	−1.538
PCDHB14	protocadherin beta 14	NM_018934	−1.536
TFPI2	tissue factor pathway inhibitor 2	NM_006528	−1.533
AKAP12	A kinase (PRKA) anchor protein 12	NM_144497	−1.529
NCOA7	nuclear receptor coactivator 7	NM_181782	−1.519

## Discussion

HIC-1 is a gene that is hypermethylated in cancer, and is commonly downregulated in human breast cancer. According to immunohistochemical studies using tissue microarrays, HIC-1 is expressed mainly in the nucleus and cytoplasm of mammary ductal epithelium. HIC-1 expression is also reduced in mammary cancer cells. In the present study, 62.5% of breast cancer samples revealed low levels of expression of HIC-1 in tissue microarray analysis. However, HIC-1 protein expression was retained in some breast carcinoma samples. There was also a trend for the downregulation of HIC-1 in older patients. Recently, Foveau et al. overexpressed HIC-1 in MDA-MB-231 breast cancer cells, which resulted in impaired cell proliferation, migration, and invasion *in vitro*. They also revealed that the tyrosine kinase receptor EphA2 is a direct target gene of HIC-1 [24]. Boulay et al. found that the β2 adrenergic receptor (ADRB2) is also a direct target of HIC-1. Consistent with this, the inactivation of HIC-1 in breast carcinoma predisposed cells to stress-induced metastasis via the up-regulation of ADRB2 [12]. To date, there is no systematic study of the precise mechanisms and molecular pathways modulated by HIC-1.

RNAa is emerging as a potential solution by using double-stranded RNA to increase endogenous gene expression. This novel technology opens a door for reactivating the expression of silenced tumor suppressor genes [15], . Several successful studies demonstrated that dsRNAs targeting promoter regions effectively restored gene expression. Chen et al. transfected saRNA targeting the p21 promoter, and induced p21 expression in T24 and J82 bladder cancer cell lines. In addition, dsP21 transfection inhibited bladder cancer cell proliferation and clonogenicity significantly [29]. Mao and colleagues induced E-cadherin expression by saRNA, which suppressed the migration and invasion of 5637 human bladder cancer cells *in vitro*. They proposed that the activation of E-cadherin by saRNA could have therapeutic benefits for bladder malignancies [30]. Huang et al. evaluated RNAa in cells derived from four mammalian species including nonhuman primates (African green monkey and chimpanzee), mice, and rats. Transfection of human saRNA into African green monkey and chimpanzee cells resulted in the induction of the target gene. The authors proposed that nonhuman primate disease models could have clinical application for validating RNAa-based drugs [23]. For example, RNAa-mediated overexpression of WT1 may have therapeutic potential in hepatocellular carcinoma [31]. Li et al. developed a 2′-fluoro-modified derivative (dsP21-322-2′F) in lipid nanoparticles, which facilitated the activation of p21 *in vivo* and led to the regression/disappearance of tumors in 40% of the treated mice [32].

Recently, we reported the reactivating efficacy of saRNAs on the tumor suppressor HIC-1 in gastric cancer. The upregulation of HIC-1 resulted in obvious anti-cancer effects [22]. Here, we screened gene expression in breast cancer, and confirmed that HIC-1 is generally downregulated in breast cancer. Next, we used RNAa to reverse HIC-1 expression in combination with 5-CdR treatment. By assessing four different dsRNAs, we identified one functional saRNA targeted to the −2998 region of the HIC-1 promoter, and revealed strong efficacy for HIC-1 expression. We next evaluated the altered expression profiles after saRNA transfection in MCF-7 and MDA-MB-231 breast cancer cells. After the re-expression of HIC-1 gene, there were 1375 differentially expressed genes between the HIC-1 activation group and control in MCF-7 cells (*P*<0.01 and fold change ≥2 or ≤−2). The upregulated genes were involved in immune activity, the inhibition of invasion, and apoptosis, whereas the downregulated genes played roles in cell migration, cell division, and cell cycle progression. For example, TIMP3, which was upregulated after HIC-1 activation, encodes metallopeptidase inhibitor 3, which inhibits matrix metalloproteinases (MMPs) in the extracellular matrix (ECM). Increased expression of MMPs was closely correlated with tumor invasion and metastasis [33−36]. CASP4 was upregulated after HIC-1 activation, which is an apoptosis-related cysteine peptidase [37], [38]. BIK, which is a BCL2-interacting killer related to apoptotic induction, was also upregulated [39−42]. The expression of BIK is known to have prognostic significance in breast cancer [43]. UBE2C/UBCH10 encodes the ubiquitin-conjugating enzyme E2C, which is downregulated after HIC-1 reactivation. Psyrri and colleagues found that elevated UBE2C mRNA expression was associated with poor disease-free and overall survival in breast cancer [44]. High tumor grade, as well as increased Ki67 protein expression, was more frequent in tumors with a high level of expression of UBE2C [45−47]. Therefore, the biological role of the growth inhibition after restoration of HIC-1 may be related partially to reduced UBE2C expression. HMMR/RHAMM (CD168) is a hyaluronan-mediated motility receptor and cell surface oncogenic protein that is commonly upregulated in human cancers. Its expression correlates well with cell motility and invasion [48−51]. Sankaran et al. reported that MTA1 (metastatic tumor antigen 1) is an upstream co-activator of HMMR expression [52]. HMMR encodes a nonintegral cell surface hyaluronan receptor and intracellular protein that promotes cell motility *in vitro* [53]. Our study revealed for the first time that HIC-1 is an upstream inhibitor of HMMR expression. CENPF is a 350/400 KDa centromere protein F (mitosin). Ueda and coworkers found that CENPF was upregulated in tumors with a high proliferation rate in breast cancer. They proposed that CENPF was a prognostic indicator for primary breast cancer [54]. Restoring the tumor suppressor function of HIC-1 gene may partially derive benefit from reduced CENPF expression on breast cancer cells. In addition, other targets such as SKA3, NTN4, IFI35, and CKS1B that were downregulated by HIC-1 activation exert important biological functions [55−67]. Chen and colleagues proposed that loss of HIC-1 function promoted tumorigenesis via the activation of the stress-controlling protein SIRT1, thereby attenuating p53 function. The inactivation of HIC-1 resulted in upregulated SIRT1 expression in normal or cancer cells [68]. Foveau and coworkers found that the tyrosine kinase receptor EphA2 was a direct target gene of HIC-1. The upregulation of EphA2 was correlated with increased cell migration [24]. However, we did not find SIRT1 or EphA2 in the list of differentially expressed genes, although the ephrin family member EFNB3 was downregulated upon HIC-1 reactivation. This may be due to the relatively limited sensitivity of the microarray. Consistent with this, we assessed the mRNA expression levels of SIRT1, EFNB3, and several apoptosis-regulating genes (BIK, CASP3, CASP4, and CASP9) in MCF-7 and MDA-MB-231 cancer cells. Both SIRT1 and EFNB3 were decreased significantly upon HIC-1 reactivation. Of the four apoptosis-regulating genes, the mRNA levels of BIK and CASP4 were increased significantly (Figure S2), as assessed by quantitative RT-PCR analysis.

Personalized targeted therapy is an upcoming trend for breast cancer treatment. Although targeted therapy for HER-2 amplification is very effective, its benefits are limited to a specific proportion of patients. Therefore, it is important to develop additional molecular targets or drugs. In present study, we successfully activated the tumor suppressor gene HIC-1 using saRNA (dsHIC1-2998) in breast cancer cells. Our results supported the hypothesis that the expression of tumor suppressor genes can be restored. Since saRNAs are small molecules, they can easily penetrate cells. For use as a gene therapy, saRNA is superior to traditional viral vector-based gene transfer. Therefore, RNAa is a potentially useful strategy for targeting specific genes. This study is the first to analyze global gene expression profiles based on HIC-1 gene reactivation, and outlines a set of important genes involved in the carcinogenesis and progression of breast cancer.

In conclusion, the findings described in the current study may provide valuable information not only for gene functional studies such as the regulation of gene expression and molecular mechanisms, but also for potential clinical applications, such as developing therapeutic drugs for breast cancer.

## Supporting Information

Figure S1 **Two-way hierarchical clustering heatmap of differentially expressed genes between HIC-1 activated MDA-MB-231 cells and control.**
(TIF)

Figure S2 **saRNAs effectively activate HIC-1 expression influence downstream genes levels in MCF-7 and MDA-MB-231 cells.** A. After 50 nM dsHIC1-2998 activation for 72 hrs on MCF-7 and MDA-MB-231 cells, the SIRT1 and EFNB3 mRNA levels were down-regulated by real-time PCR. Relative to a value of 1 for mRNA expression of SIRT1 by mock, the relative mRNA expressions of HIC-1 were 0.679. Relative to a value of 1 for mRNA expression of EFNB3 by mock, the relative mRNA expressions of HIC-1 was 0.427 in MCF-7 cells. While, Relative to a value of 1 for mRNA expression of SIRT1 by mock, the relative mRNA expressions of HIC-1 were 0.537. Relative to a value of 1 for mRNA expression of EFNB3 by mock, the relative mRNA expressions of HIC-1 was 0.466 in MDA-MB-231 cells. B. After 50 nM dsHIC1-2998 activation for 72 hrs on MCF-7 and MDA-MB-231 cells, The mRNA levels of BIK, CASP3, CASP4 and CASP9 were assayed by real-time PCR. Relative to control, the mRNA levels of BIK increased 2.854-fold, and the mRNA levels of CASP4 increased 1.533-fold in MCF-7 cells. But the mRNA levels of CASP3 and CASP9 did not change obviously. In MDA-MB-231 cells, relative to control, the mRNA levels of BIK increased 2.906-fold, and the mRNA levels of CASP4 increased 2.090-fold. But the mRNA levels of CASP3 and CASP9 did not change obviously.
(TIF)
