# Supplementary figures and images for: Small Activating RNA Restores the Activity of the Tumor Suppressor HIC-1 on Breast Cancer
Source: PLoS One. 2014 Jan 28;9(1):e86486. doi: 10.1371/journal.pone.0086486 (PMC3904905; doi:10.1371/journal.pone.0086486)

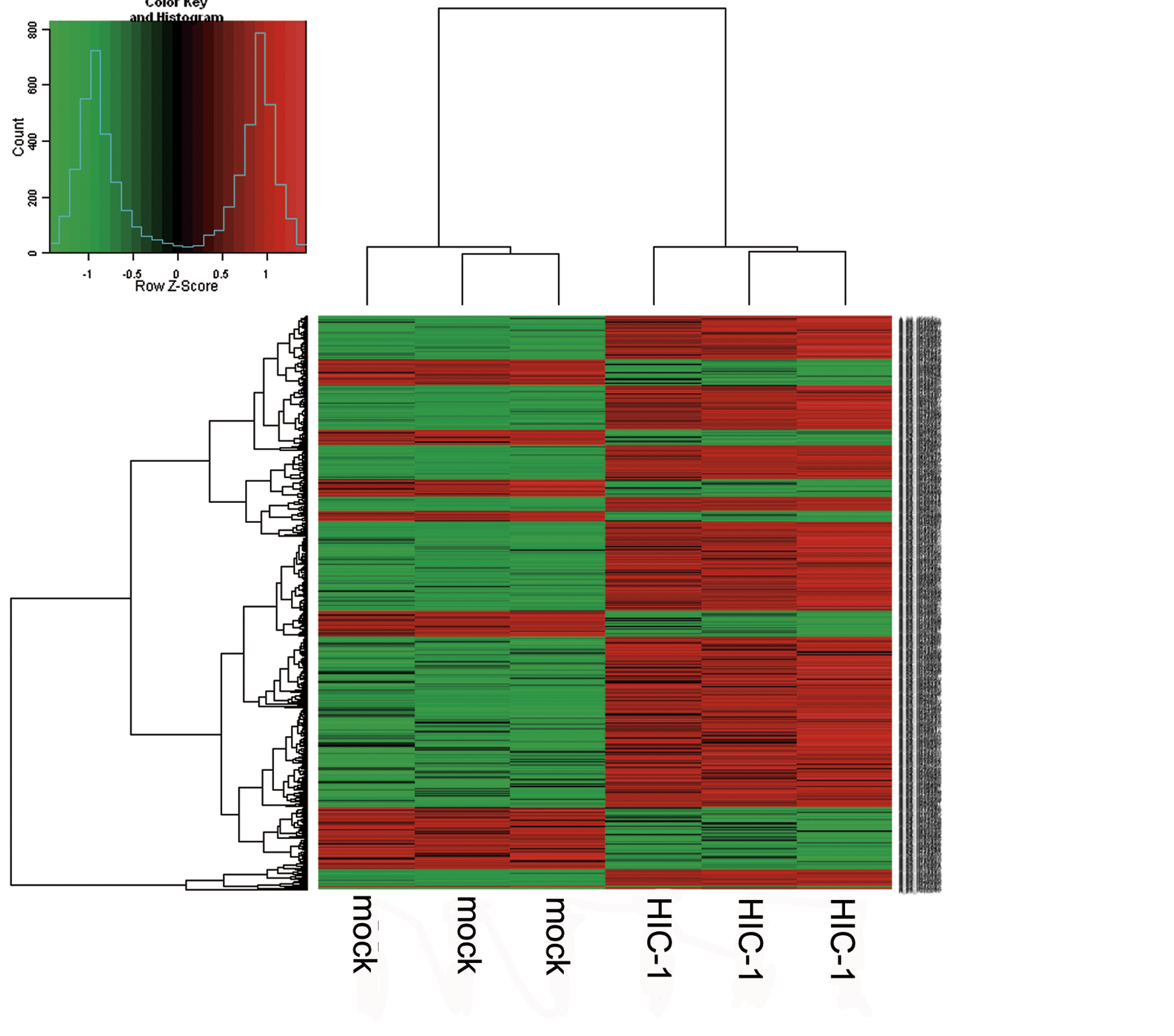

Supplement: Figure S1 — Two-way hierarchical clustering heatmap of differentially expressed genes between HIC-1 activated MDA-MB-231 cells and control. (TIF) [file pone.0086486.s001.tif]

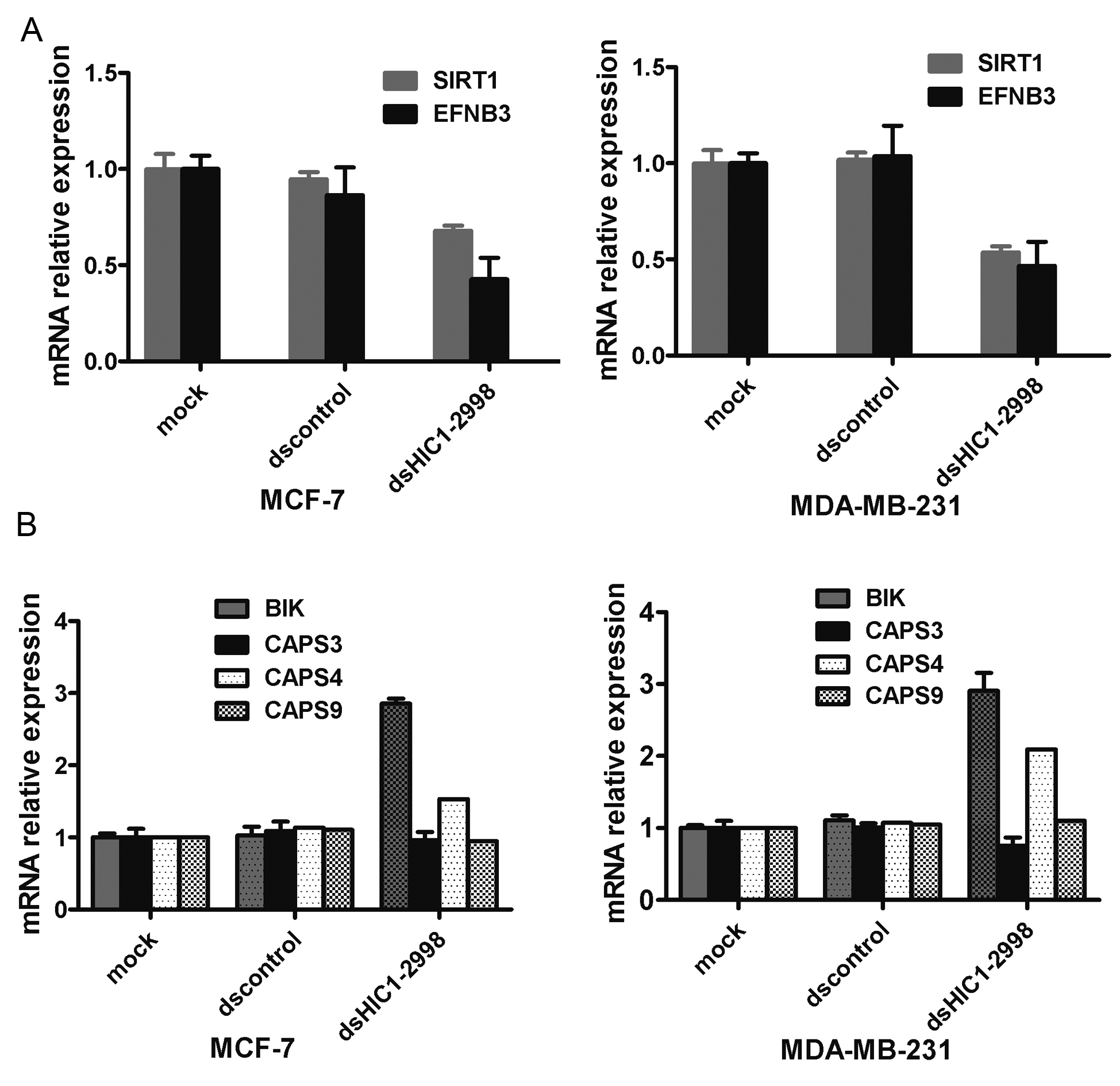

Supplement: Figure S2 — saRNAs effectively activate HIC-1 expression influence downstream genes levels in MCF-7 and MDA-MB-231 cells. A. After 50 nM dsHIC1-2998 activation for 72 hrs on MCF-7 and MDA-MB-231 cells, the SIRT1 and EFNB3 mRNA levels were down-regulated by real-time PCR. Relative to a value of 1 for mRNA expression of SIRT1 by mock, the relative mRNA expressions of HIC-1 were 0.679. Relative to a value of 1 for mRNA expression of EFNB3 by mock, the relative mRNA expressions of HIC-1 was 0.427 in MCF-7 cells. While, Relative to a value of 1 for mRNA expression of SIRT1 by mock, the relative mRNA expressions of HIC-1 were 0.537. Relative to a value of 1 for mRNA expression of EFNB3 by mock, the relative mRNA expressions of HIC-1 was 0.466 in MDA-MB-231 cells. B. After 50 nM dsHIC1-2998 activation for 72 hrs on MCF-7 and MDA-MB-231 cells, The mRNA levels of BIK, CASP3, CASP4 and CASP9 were assayed by real-time PCR. Relative to control, the mRNA levels of BIK increased 2.854-fold, and the mRNA levels of CASP4 increased 1.533-fold in MCF-7 cells. But the mRNA levels of CASP3 and CASP9 did not change obviously. In MDA-MB-231 cells, relative to control, the mRNA levels of BIK increased 2.906-fold, and the mRNA levels of CASP4 increased 2.090-fold. But the mRNA levels of CASP3 and CASP9 did not change obviously. (TIF) [file pone.0086486.s002.tif]
